# Trends in the incidence of AIDS and epidemiological features in Tianjin, China from 2005 to 2016

**DOI:** 10.18632/oncotarget.21016

**Published:** 2017-09-18

**Authors:** Ping Ma, Liying Gao, Defa Zhang, Aiping Yu, Chunting Qiu, Lei Li, Fangfang Yu, Yue Wu, Wei You, Yanyun Guo, Xianjia Ning, Wei Lu

**Affiliations:** ^1^ Department of Infectious Disease, Tianjin Second People's Hospital, Tianjin 300192, China; ^2^ Center of Epidemiology and Department of Neurology, Tianjin Medical University General Hospital, Tianjin 300052, China; ^3^ Tianjin Neurological Institute, Key Laboratory of Post-Neuroinjury Neuro-repair and Regeneration in Central Nervous System, Ministry of Education and Tianjin City, Tianjin 300052, China; ^4^ Department of Epidemiology, Tianjin Neurological Institute, Tianjin 300052, China

**Keywords:** AIDS, epidemiology, incidence, trends, China

## Abstract

The objective of this study was to assess the epidemiological trends among patients with AIDS in Tianjin, China. A long-term surveillance study was conducted from 2005 to 2016 in Tianjin, China. All patients with AIDS registered in Tianjin from 2005 to 2016 were recruited to this study. Demographic information and clinical features were recorded. A total of 3062 patients with AIDS who were treated with antiretroviral therapy were included in this study. Among AIDS patients, men were more likely to be younger than women (age, 37.84 years vs. 43.27 years; *P* < 0.001). The incidence of AIDS increased by 39.6% annually over the past 12 years overall. There was the greatest increase (by 44.7%) for homosexual route. Moreover, the proportion of patients aged < 30 years increased considerably over the 12-year study period, while there was a decrease in the proportion of patients aged ≥ 35 years. The frequency of homosexual transmission increased by 86% from before 2011 to 2016, but the frequency of heterosexual transmission decreased by 49%. The frequency of transmission through intravenous drug use decreased in men and patients aged 25–29 years. For those infected through homosexual transmission, there was a significant increase in the numbers of patients aged 20–24 years and 25–29 years. It is important for developing countries to effectively prevent and control the transmission of HIV/AIDS; in particular, it is crucial to promote disease education and sexual protection among young men.

## INTRODUCTION

Acquired immune deficiency syndrome (AIDS) has recently become the leading cause of death, and the number of AIDS-related deaths is significantly higher than the number of deaths due to any other infectious disease in China [1].

Global human immunodeficiency virus (HIV) incidence reached its peak in 1997, at 3.3 million new infections (95% uncertainty interval [UI] 3.1–3.4 million). Annual incidence has stayed relatively constant at about 2.6 million cases per year (from 2.5 to 2.8 million) since 2005, after a period of fast decline by 4.8% (4.0–5.5) per year between 1997 and 2005. The number of people living with HIV/AIDS has been steadily increasing and reached 38.8 million (95% UI 37.6–40.4 million) in 2015. At the same time, HIV/AIDS mortality has been declining at a steady pace, from a peak of 1.8 million deaths (95% UI 1.7–1.9 million) in 2005, to 1.2 million deaths (1.1–1.3 million) in 2015. At the end of 2015, there were 55,200 (95% UI 39,820–75,980) new cases of HIV infection in China; furthermore, 779,480 (95% UI 573,770–1,024,760) people were living with HIV/AIDS [2]. However, the age-standardized prevalence of HIV/AIDS increased by 4% (from 3% to 5%) annually during 2005 to 2015, and the mortality rate increased by 8% (from 7% to 9%) in China [2]. More than 17 million individuals are receiving treatment worldwide, but about 20 million HIV-infected individuals are still untreated [3].

A rapid increase in the numbers of HIV/AIDS cases has been reported in previous studies conducted in Iran, Japan, and China [4–6]. From 2010 to 2014, reported surviving cases and deaths from AIDS increased continuously along with the increase in numbers of detected cases and cases treated with antiretroviral therapy (ART); at the end of 2014, there were a total of 205,000 people living with AIDS and 159,000 AIDS-related deaths in China [7]. Furthermore, the epidemiologic characteristics of HIV/AIDS vary among different areas in China [8]. By the end of 2014, twelve provinces with the highest HIV prevalence accounted for 83.5% of all cases in China, whereas nine provinces with the lowest HIV prevalence only accounted for 3.4% of all cases [7]. A large number of studies have reported the prevalence of AIDS in the areas with the highest HIV prevalence [9–14], though few have investigated incidence in the areas with the lowest HIV prevalence [15, 16]. Moreover, studies on the trends in the clinical epidemiology of HIV/AIDS following rapid economic development from representative samples in China are scarce, especially in developed regions.

There were two primary paths to monitor and manage individuals living with HIV/AIDS, which included hospital-based/clinic-based surveillance and sentinel surveillance [17–19]. The official surveillance data that integrated information from both surveillance systems was initiated in 2005 [19–21]. Thus, we aimed to explore the latest long-term trends in epidemiological features from 2005 to 2016, during a period of rapid economic development, among individuals living with AIDS in Tianjin, China.

## RESULTS

### Descriptive characteristics

A total of 3063 AIDS patients were registered in the Tianjin Second People's Hospital from January 2005 to December 2016. Finally, 3062 cases were assessed in this study after excluding 1 case with vertical transmission. Men accounted for 93.6% of cases (2867 cases), and women accounted for 6.4% (195 cases), with an overall mean age of 38.19 years; men were younger than women (37.84 years vs. 43.27 years; *P* < 0.001). There were higher frequencies of patients aged 25–29 years and 30–34 years in men, but in women, more patients were aged ≥ 50 years. Men were more likely to be infected through homosexual intercourse (68.3%), while women were more likely to be infected through heterosexual intercourse (71.3%; Table 1).

**Table 1:** The demographical characteristics of participants in this study

Categories	Men	Women	Total
Number, n (%)	2867 (93.6)	195 (6.4)	3062
Age, year, means (SD)	37.84 (11.79)	43.27 (12.61)	38.19 (11.91)
Age group, n (%)			
< 20	27 (0.9)	3 (1.5)	30 (1.0)
20~	294 (10.3)	7 (3.6)	301 (9.8)
25~	560 (19.5)	20 (10.3)	580 (18.9)
30~	553 (19.3)	21 (10.8)	574 (18.7)
35~	338 (11.8)	35 (17.9)	373 (12.2)
40~	283 (9.9)	23 (11.8)	306 (10.0)
45~	286 (10.0)	32 (16.4)	318 (10.4)
≥ 50	526 (18.3)	54 (27.7)	580 (18.9)
Route of infection			
Transfusion/Blood sample	37 (1.3)	5 (2.6)	42 (1.4)
Intravenous drug using	116 (4.0)	18 (9.2)	134 (4.4)
Homosexual	1957 (68.3)	2 (1.0)	1959 (64.0)
Heterosexual	472 (16.5)	139 (71.3)	611 (20.0)
Unknown	285 (9.9)	31 (15.9)	316 (10.3)

### Trends in the incidence of AIDS by transmission route

Table 2 shows that incidence increased by 39.6% (34.7%–44.4%) annually overall. There was a significant increase in incidence over the 12-year period, with an annual percent changes of 39.6% overall, 30% for those infected through intravenous drug use (IDU), 44.7% for those infected through homosexual intercourse, 35% for those infected through heterosexual intercourse, and 34.8% for those infected through an unknown transmission route (all *P* < 0.001). However, there was no significant change in infection through transfusion (*P* = 0.061).

**Table 2:** Trends in the incidence of AIDS by transmission route during 2005 to 2016 in Tianjin, China (1/100000 population)

Year	Total	Transfusion	IDU	Homosexual	Heterosexual	Unknown
2005	0.13 (0.06, 0.20)	0	0.01 (0, 0.38)	0.04 (0, 0.08)	0.08 (0.02, 0.14)	0
2006	0.15 (0.07, 0.23)	0.03 (0, 0.06)	0.03 (0, 0.06)	0.08 (0.02, 0.13)	0.03 (0, 0.06)	0
2007	0.18 (0.10, 0.26)	0	0.04 (0, 0.08)	0.10 (0.03, 0.16)	0.02 (0, 0.05)	0.02 (0.01, 0.06)
2008	0.29 (0.20, 0.38)	0	0.04 (0, 0.09)	0.13 (0.06, 0.20)	0.10 (0.03, 0.16)	0.11 (0.04, 0.18)
2009	0.56 (0.46, 0.66)	0.01 (0, 0.03)	0.04 (0, 0.08)	0.30 (0.21, 0.39)	0.09 (0.03, 0.16)	0.11 (0.05, 0.18)
2010	0.94 (0.89, 0.99)	0.01 (0.01, 0.02)	0.06 (0.01, 0.10)	0.51 (0.42, 0.61)	0.34 (0.24, 0.44)	0.02 (0.01, 0.05)
2011	1.87 (1.06, 2.68)	0	0.14 (0.07, 0.20)	1.14 (1.08, 1.77)	0.56 (0.47, 0.65)	0.37 (0.25, 0.48)
2012	2.32 (2.23, 2.41)	0	0.15 (0.08, 0.21)	1.61 (1.38, 1.84)	0.51 (0.26, 0.76)	0.05 (0.01, 0.09)
2013	2.64 (2.55, 2.73)	0.05 (0.01, 0.09)	0.19 (0.12, 0.26)	1.43 (1.22, 1.64)	0.52 (0.43, 0.61)	0.45 (0.33, 0.67)
2014	4.62 (4.50, 4.74)	0.06 (0.02, 0.11)	0.25 (0.17, 0.32)	3.03 (3.00, 3.61)	0.75 (0.66, 0.82)	0.54 (0.41, 0.66)
2015	5.83 (5.70, 5.96)	0.13 (0.07, 0.19)	0.14 (0.08, 0.20)	3.89 (3.55, 4.23)	1.12 (0.93, 1.14)	0.55 (0.42, 0.68)
2016	5.68 (5.55, 5.81)	0.05 (0.02, 0.09)	0.77 (0.70, 0.84)	3.79 (3.45, 4.13)	1.00 (0.82, 1.17)	0.76 (0.61, 0.91)
Trends^*^, %	39.6 (34.7, 44.4)	19.7 (-1.4, 40.7)	30.0 (23.1, 37.0)	44.7 (39.0, 50.3)	35.0 (24.0, 46.0)	34.8 (11.6, 58.1)
P	< 0.001	0.061	< 0.001	< 0.001	< 0.001	0.009

IDU: intravenous drug using; ^*^ presented as annual rate of change.

**Figure 1:**
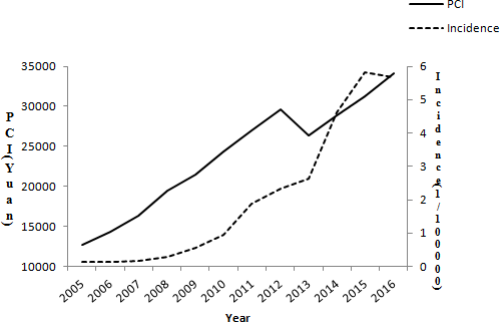
The incidence of AIDS following the rapid growth of per capita disposable income in Tianjin from 2005 to 2016

The incidence of AIDS in Tianjin increased significantly from 2005 to 2016 following the rapid growth of per capita disposable income (Figure 1).

### Trends in epidemiological features

Compared to male patients, there was a significant decrease in the prevalence of female patients (from 15.9% before 2011 to 4.5% in 2016; *P* < 0.001). The overall mean age of patients was nearly 12 years lower in 2016 than before 2011 (34.78 years vs. 46.43 years; *P* < 0.001). Moreover, the proportion of patients in the < 30-year age groups, including those aged < 20 years, 20–24 years, and 25–29 years, was remarkably increased over the study period, with an odds ratio (95% CI) of 26.67 (11.69, 60.82; *P* < 0.001) for those aged < 30 years. Simultaneously, the frequency of individuals aged ≥ 35 years decreased significantly from 2005 to 2016. Furthermore, there were obvious decreases in the frequencies of transmission through IDU and heterosexual intercourse, while there were increases in the frequencies of transmission through homosexual intercourse and unknown transmission routes. Compared to before 2011, the frequency of homosexual transmission increased by 86% in 2016, with a relative risk (RR; 95% confidence interval [CI]) of 1.86 (1.37, 2.53; *P* < 0.001); however, the frequency of heterosexual transmission decreased by 49%, with an RR (95% CI) of 0.51 (0.36, 0.72; *P* < 0.001; Table 3).

**Table 3:** Trends in the epidemiological features among patients with AIDS during 2005 to 2016 in Tianjin, China

Categories	Before 2011	2011	2012	2013	2014	2015	2016	*P*
Cases, n (%)	214 (7.0)	204 (6.7)	267 (8.7)	319 (10.4)	577 (18.8)	745 (24.3)	736 (24.0)	—
Women	34 (15.9)	16 (7.8)	22 (8.2)	34 (10.7)	19 (3.3)	37 (5.0)	33 (4.5)	< 0.001
Death, n (%)	10 (4.7)	13 (6.4)	10 (3.7)	8 (2.5)	12 (2.1)	10 (1.3)	8 (1.1)	< 0.001
Age, years, mean (SD)	46.43 (10.23)	43.51 (11.24)	40.42 (10.99)	40.90 (11.52)	37.72 (11.50)	36.56 (11.65)	34.78 (11.42)	< 0.001
Age group, n (%)								< 0.001
< 20 years	0	0	1 (0.4)	1 (0.3)	2 (0.3)	9 (1.2)	17 (2.3)	< 0.001
20 years ~	1 (0.5)	5 (2.5)	7 (2.6)	11 (3.4)	55 (9.5)	95 (12.8)	127 (17.3)	< 0.001
25 years ~	5 (2.3)	14 (6.9)	55 (20.6)	46 (14.4)	119 (20.6)	165 (22.1)	176 (23.9)	< 0.001
30 years ~	24 (11.2)	37 (18.1)	43 (16.1)	60 (18.8)	127 (22.0)	144 (19.3)	139 (18.9)	0.026
35 years ~	32 (15.0)	33 (16.2)	33 (12.4)	49 (15.4)	62 (10.7)	93 (12.5)	71 (9.6)	0.005
40 years ~	39 (18.2)	25 (12.3)	32 (12.0)	37 (11.6)	54 (9.4)	60 (8.1)	59 (8.0)	< 0.001
45 years ~	41 (19.2)	31 (15.2)	34 (12.7)	37 (11.6)	58 (10.1)	64 (8.6)	53 (7.2)	< 0.001
≥ 50 years	72 (33.6)	59 (28.9)	62 (23.2)	78 (24.5)	100 (17.3)	115 (15.4)	94 (12.8)	< 0.001
Route of infection								
Transfusion	4 (1.9)	0	0	6 (1.9)	8 (1.4)	19 (2.2)	7 (1.0)	0.378
IDU	20 (9.3)	15 (7.4)	17 (6.4)	23 (7.2)	31 (5.4)	18 (2.1)	10 (1.4)	< 0.001
Homosexual	111 (51.9)	124 (60.8)	185 (69.3)	173 (54.2)	378 (65.5)	575 (65.6)	491 (66.7)	< 0.001
Heterosexual	63 (29.4)	61 (29.9)	59 (22.1)	63 (19.7)	93 (16.1)	166 (18.9)	129 (17.5)	< 0.001
Unknown	16 (7.5)	4 (2.0)	6 (2.2)	54 (16.9)	67 (11.6)	98 (11.2)	99 (13.5)	< 0.001

### Trends in transmission routes

Table 4 shows that the frequency of transmission through IDU decreased in men and individuals aged 25–29 years, with odds ratios (95% CIs) of 0.15 (0.07, 0.36) and 0.02 (0.00, 0.43), respectively.

**Table 4:** Trends in the infected routes among male and young patients with AIDS during 2005 to 2016 in Tianjin, China

Categories	Before 2011	2011	2012	2013	2014	2015	2016	*P*
Men:								< 0.001
Transfusion	4 (2.2)	0	0	4 (1.4)	7 (1.3)	16 (2.3)	6 (0.9)	
IDU	14 (7.8)	14 (7.4)	15 (6.1)	21 (7.4)	29 (5.2)	14 (2.0)	9 (1.3)	
Homosexual	111 (61.7)	124 (66.0)	185 (75.5)	171 (60.0)	378 (67.7)	497 (70.2)	491 (69.8)	
Heterosexual	37 (20.6)	46 (24.5)	39 (15.9)	44 (15.4)	79 (14.2)	118 (16.7)	109 (15.5)	
Unknown	14 (7.8)	4 (2.1)	6 (2.4)	45 (15.8)	65 (11.6)	63 (8.9)	88 (12.5)	
< 20 years:								0.697
Transfusion	0	0	0	0	0	0	0	
IDU	0	0	0	0	0	0	0	
Homosexual	0	0	0	1 (100.0)	2 (100.0)	9 (100.0)	13 (76.5)	
Heterosexual	0	0	1 (100.0)	0	0	0	1 (5.9)	
Unknown	0	0	0	0	0	0	3 (17.6)	
20 years ~								0.775
Transfusion	0	0	0	0	0	1 (1.1)	1 (0.8)	
IDU	0	0	2 (28.6)	1 (9.1)	2 (3.6)	1 (1.1)	3 (2.4)	
Homosexual	1 (100.0)	4 (80.0)	3 (42.9)	5 (45.5)	44 (80.0)	80 (84.2)	101 (79.5)	
Heterosexual	0	1 (20.0)	2 (28.6)	3 (27.3)	3 (5.5)	7 (7.4)	11 (8.7)	
Unknown	0	0	0	2 (18.2)	6 (10.9)	6 (6.3)	11 (8.7)	
25 years~								0.020
Transfusion	0	0	0	0	1 (0.8)	2 (1.2)	0	—
IDU	1 (20.0)	0	3 (5.5)	3 (6.5)	10 (8.4)	2 (1.2)	1 (0.6)	0.002
Homosexual	3 (60.0)	11 (78.6)	45 (81.8)	27 (58.7)	91 (76.5)	122 (73.9)	132 (75.0)	0.904
Heterosexual	1 (20.0)	2 (14.3)	7 (12.7)	5 (10.9)	10 (8.4)	29 (17.6)	25 (14.2)	0.433
Unknown	0	1 (7.1)	0	11 (23.9)	7 (5.9)	10 (6.1)	18 (10.2)	0.489
< 30 years:								0.037
Transfusion	0	0	0	0	1 (0.6)	3 (1.1)	1 (0.3)	—
IDU	1 (16.7)	0	5 (7.9)	4 (6.9)	12 (6.8)	3 (1.1)	4 (1.3)	< 0.001
Homosexual	4 (66.7)	15 (78.9)	48 (76.2)	33 (56.9)	137 (77.8)	211 (78.4)	246 (76.9)	0.199
Heterosexual	1 (16.7)	3 (15.8)	10 (15.9)	8 (13.8)	13 (7.4)	36 (13.4)	37 (11.6)	0.578
Unknown	0	1 (5.3)	0	13 (22.4)	13 (7.4)	16 (5.9)	32 (10.0)	0.329

IDU: intravenous drug using.

With regard to infection through homosexual transmission, there was no difference in frequencies between men and women. However, there was a significant increase in those aged 20–24 years and 25–29 years, with increases of 27.5-fold and 12.2-fold, respectively (Table 5).

**Table 5:** Trends in the epidemiological features among AIDS patients with infected by homosexual during 2005 to 2016 in Tianjin, China

Categories	Before 2011	2011	2012	2013	2014	2015	2016	P
Gender:								0.401
Men	111 (100.0)	124 (100.0)	185 (100.0)	171 (98.8)	378 (100.0)	497 (100.0)	491 (100.0)	
Women	0	0	0	2 (1.2)	0	0	0	
Age, years, mean (SD)	45.50 (11.01)	42.52 (11.10)	39.64 (10.85)	40.80 (11.91)	35.53 (10.34)	34.63 (10.59)	32.49 (9.87)	< 0.001
Age group:								< 0.001
< 20 years:	0	0	0	1 (0.6)	2 (0.5)	9 (1.8)	13 (2.6)	< 0.001
20 years ~	1 (0.9)	4 (3.2)	3 (1.6)	5 (2.9)	44 (11.6)	80 (16.1)	101 (20.6)	< 0.001
25 years~	3 (2.7)	11 (8.9)	45 (24.3)	27 (15.6)	91 (24.1)	122 (24.5)	132 (26.9)	< 0.001
30 years ~	18 (16.2)	21 (16.9)	36 (19.5)	28 (16.2)	93 (24.6)	90 (18.1)	101 (20.6)	0.291
35 years ~	16 (14.4)	23 (18.5)	19 (10.3)	27 (15.6)	37 (9.8)	61 (12.3)	42 (8.6)	0.008
40 years ~	17 (15.3)	15 (12.1)	21 (11.4)	21 (12.1)	30 (7.9)	47 (9.5)	34 (6.9)	0.002
45 years ~	22 (19.8)	18 (14.5)	20 (10.8)	24 (13.9)	38 (10.1)	34 (6.8)	31 (6.3)	< 0.001
≥ 50 years	34 (30.6)	32 (25.8)	41 (22.2)	40 (23.1)	43 (11.4)	54 (10.9)	37 (7.5)	< 0.001

## DISCUSSION

This is the first study to assess the trends in the epidemiological features of AIDS patients in Tianjin, China. The study indicated that the incidence of AIDS increased significantly from 2005 to 2016 following rapid economic development. The incidence of AIDS increased 39.6% annually overall; the frequency of infection through all transmission routes, except for transfusion, increased significantly. Male patients were more common than female patients in this developed region, and the mean age of men was 12 years younger than that of women. Homosexual transmission was the dominant infection route in male AIDS patients (68.3%), while women were more likely to be infected through heterosexual transmission (71.3%). Over the 12-year study period, there were significant increases in the proportion of individuals aged < 30 years and the frequency of transmission through homosexual intercourse, while there were decreases in the number of female patients, number of patients aged ≥ 35 years, frequency of transmission through IDU, and frequency of transmission through heterosexual intercourse. The frequency of homosexual transmission increased by 86% in 2016 compared to before 2011, but the frequency of heterosexual transmission decreased by 49%. The frequency of those infected through homosexual transmission increased by 27.5-fold in patients aged 20–24 years and 12.2-fold in those aged 25–29 years.

Since the introduction of effective combination ART, the annual rate of new AIDS diagnoses has decreased by more than half in the United States [22, 23]. About 1.2 million individuals were living with HIV in the United States in 2012 [24]. Over 500,000 people living with HIV have ever received the diagnosis of AIDS, and about 27,000 individuals are newly diagnosed with AIDS each year in the United States [23]. Up-to-date research results showed that although the incidence substantially declined by 2% globally from 2005 to 2015, the incidence rates increased in 74 countries [25]. There was a marginal decrease in the age-standardized incidence of HIV/AIDS by 1% (0–4%) in China [25]. However, the number of surviving individuals infected with HIV and diagnosed with AIDS has continued to persistently increase [7]. In contrary to previous reports, the incidence of AIDS was dramatically elevated in the present study, although the incidence of AIDS remained low overall. The annual rate of change was 39.6% from 2005 to 2016.

IDU is the most common route of HIV transmission in men in Iran, accounting for approximately 70% of established transmissions [16]. Meanwhile, a study in the United States reported the most common HIV transmission route was homosexual contact in men, whereas IDU ranked third [26]. A previous study conducted in 2008 demonstrated that IDU was the most common HIV transmission route in China, accounting for > 43% of cases [27]. Along with economic growth in China, there were remarkable changes in HIV transmission routes, transitioning from mainly IDU, blood transfusion, and plasma donation to sexual transmission. A significant increase in HIV infection has been observed among men who have sex with men (MSM), especially in economically developed areas. The percentage of HIV infection mediated by MSM behavior has increased annually, and has currently become the major route of infection in male patients [28]. HIV epidemics among MSM have occurred more recently than epidemics among intravenous drug users and female sex workers in China. In four major Chinese municipalities (including Beijing, Tianjin, Shanghai, and Chongqing), HIV prevalence in MSM was greater than 5% in 2010 [29]. In line with these reports, in this study, we found that homosexual transmission was the dominant infection route in male AIDS patients (accounting for 68.3%), while women were more likely to be infected through heterosexual transmission (accounting for 71.3%). Over the past 12 years, the incidence of homosexual AIDS transmission increased by 44.7% annually. The frequency of homosexual transmission increased by 86% in 2016 compared to the frequency before 2011. However, there were significant decreases in the frequencies of transmission through IDU and heterosexual intercourse; the frequency of infection through heterosexual transmission decreased by 49%. Tianjin is one of the developed super cities in China. Rapid economic development and population mobility, especially accelerated urbanization, have increased the population at risk of HIV/AIDS infection. Thus, it is important to promote disease education and sexual protection among young men. Simultaneously, we should also promote safe sex among women to prevent increases in the incidence of AIDS in China.

Many studies have demonstrated that the prevalence of HIV/AIDS is higher in men than in women. However, the 2010 Joint United Nations Programme on HIV/AIDS Global Report stated that more than 50% of people living with HIV worldwide are female [30]. The frequency of male individuals living with HIV/AIDS has been reported to be two-fold higher than the frequency of female individuals living with HIV/AIDS [31]. In this study, women were more likely to be infected through heterosexual transmission (71.3%). Over the 12-year study period, there was a significant decrease in the number of female patients. This disparity according to sex among individuals living with HIV/AIDS may be explained by the transition of infection routes. MSM continued to account for a large percentage (61%) of new HIV infections in the United States in 2009 [32]. HIV infections in MSM have been increasing at an alarming annual rate of 8% since 2001 [33]. Various studies during the past decade have assessed the severity of the HIV epidemic among MSM in low-income and middle-income countries [34, 35]. The prevalence of HIV among MSM in developing Asian countries was high and increasing, with rates of 9.0% in Indonesia and 14.7% in India [34]. Studies in Thailand showed that HIV prevalence among MSM increased rapidly form 17.3% in 2003 to 30.8% in 2007 [36].

AIDS was the leading cause of death among men and women aged 25 to 44 years and the eighth most common cause of death worldwide [26]. A previous study indicated that the mean ages of female and male patients were 44 years and 49 years, respectively [31]. Although HIV infection affects less than 1% of the general population in China, the average age of patients is 37 years (range, 22 to 63 years) [37]. Moreover, among MSM with AIDS, approximately 80% were aged < 30 years in China [38].

Consistent with this research, there was a mean age of 38.19 years overall in this study, with a more than a 5-year difference between men and women. Over the study period, there was a significant increase in the proportion of individuals aged < 30 years, but a decrease in the proportion of those aged ≥ 35 years. Moreover, the proportion of patients aged 20–24 years and 25–29 years among those infected through homosexual transmission significantly increased by 27.5-fold and 12.2-fold, respectively. However, the frequency of those aged ≥ 35 years infected through homosexual transmission decreased remarkably. The rapid increase in the rate of homosexual transmission may be due to the younger average age in Tianjin, China over the past decade.

There were several limitations in the present study. First, data were obtained from a northern city in China, and may not be considered a national representative sample. However, Tianjin is located in a developed area in China, and the transition in the epidemiology of AIDS may represent trends in other economically developed areas in China. Second, information on education level, marital status, and spouses were not assessed in this study due to individual privacy. Finally, the surveillance of individuals living with HIV/AIDS early in the study period may have been underrated. However, Tianjin Second People's Hospital is the only qualified sentinel hospital in Tianjin, China. All data involved in this study were obtained from the official registry and are reliable. However, a few patients diagnosed with AIDS were not included in this study either for personal reasons or because they did not receive ART. Thus, actually, the real incidence of AIDS in the general population may be much higher than the findings reported in this study.

This study is the first to demonstrate the epidemiological transition of HIV/AIDS in Tianjin, China. The study indicated that the incidence of AIDS increased significantly from 2005 to 2016 following the rapid economic development in Tianjin, China. The incidence of AIDS increased 39.6% annually; the greatest change was observed in the frequency of homosexual transmission (increased by 44.7% annually). Young men were more the predominant demographic in this developed region, with the dominant infection route of homosexual transmission. Over the past 12 years, there were significant increases in the frequencies of individuals aged < 30 years and homosexual transmission, and decreases in the frequencies of female patients, those aged 35 years and over, and transmission through IDU and heterosexual intercourse. Tianjin is one of the developed super cities in China. Rapid economic development and population mobility, especially accelerated urbanization, likely resulted in an increase in the population at risk of HIV/AIDS infection. These findings suggest that crucial efforts are needed to promote disease education and safe sex to prevent increases in AIDS incidence in China. In particular, it is important for developing countries to effectively prevent and control the transmission of HIV/AIDS worldwide.

## MATERIALS AND METHODS

### Subjects

All consecutive newly diagnosed AIDS patients treated with ART in the Department of Infection, Tianjin Second People's Hospital from January 2005 to December 2016 were recruited to this study. This is the only designated hospital to receive AIDS patients in Tianjin, China.

The study design and protocol were approved by the ethics committee of Tianjin Second People's Hospital, and written informed consent was obtained from each participant.

### Data collection

Information on demographics (including sex and age) and disease characteristics (including date of AIDS diagnosis, date of ART initiation, and infection routes) were collected. Treatment with ART was recorded during the periods of study. Moreover, the levels of CD4 and viral load on admission and follow-up were measured.

### Age, transmission, and study period classifications

Age was categorized into eight groups: < 20 years, 20–24 years, 25–29 years, 30–34 years, 35–39 years, 40–44 years, 45–49 years, and ≥ 50 years. Infectious route was categorized into five groups according to patient self-reporting; categories included transfusion/blood donation, IDU, homosexual intercourse, heterosexual intercourse, and unknown. This study was performed starting in 2005, but the years before 2011 were combined due to the limited number of AIDS cases from 2005 to 2010. Thus, trends in the epidemiology of AIDS were analyzed according to the following study period categories: 2005–2010, 2011, 2012, 2013, 2014, 2015, and 2016.

### Calculation of annual AIDS incidence

The incidence of AIDS on a yearly basis during 2005 to 2016 was calculated using annual registry cases divided by the annual population in Tianjin, and is presented as 1/100,000 population.

### Per capita disposable income (PCI)

PCI determines an individual's ability to purchase goods or services. It is calculated by taking income earned from all sources (wages, government transfers, rental income, etc.) minus taxes, savings, and some non-tax payments (e.g., fines, forfeitures, and donations) and dividing by the total population [39].

### Statistical analysis

Age is presented as means with standard deviations by sex or by year, and was compared between men and women using the Student's *t*-test or between years using analysis of variance. Categorized variables are presented as numbers of cases (rates), and were compared by sex or years using the chi-squared test for trends. We estimated the RR according to sex, age group, and transmission route during the study periods using Poisson regression, with the first period as the reference; results are presented as RRs with 95% CIs. Trends in the incidence of AIDS are expressed as the annual percent change, using the regression model log (rt) = a + bt, where log denoted the natural logarithm and t was the year [40]. The trend b was estimated from ordinary regression, and 100b represented the estimated annual percent change in incidence. Correlation analysis was performed to analyze the relationship between PCI and incidence of AIDS, and results are presented using correlation coefficients. All statistical analyses were performed using SPSS version 19.0 (SPSS Inc., Chicago, IL), and a two-tailed *P* value < 0.05 indicated statistical significance.

## Abbreviations

AIDS: Acquired Immune Deficiency Syndrome
HIV: human immunodeficiency virus
UI: uncertainty interval
ART: antiretroviral therapy
IDU: Intravenous drug use
MSM: sex with men
RRs: relative risks
CIs: confidence intervals
